# Identification of Key Pathways and Genes Related to the Development of Hair Follicle Cycle in Cashmere Goats

**DOI:** 10.3390/genes12020180

**Published:** 2021-01-27

**Authors:** Jianfang Wang, Jie Sui, Chao Mao, Xiaorui Li, Xingyi Chen, Chengcheng Liang, Xiaohui Wang, Si-Hu Wang, Cunling Jia

**Affiliations:** College of Animal Science and Technology, Northwest A&F University, Yangling 712100, Shaanxi, China; jfwang@nwsuaf.edu.cn (J.W.); suijie_on@163.com (J.S.); mc@nwafu.edu.cn (C.M.); lixiaorui012@163.com (X.L.); chenxingyi@nwafu.edu.cn (X.C.); lcc20151120@nwafu.edu.cn (C.L.); wangxh3607@nwafu.edu.cn (X.W.); sihumeng@126.com (S.-H.W.)

**Keywords:** hair follicle cycle, WGCNA, coexpression network, hub genes

## Abstract

The development of hair follicle in cashmere goats shows significant periodic change, as with mice and humans. However, for cashmere goat with double-coat, the periodic change may be due to other regulatory molecules and signal pathways. To understand the mechanism of periodic development of hair follicle, we performed a weighted gene coexpression network analysis (WGCNA) to mine key genes and establish an interaction network by utilizing the NCBI public dataset. Ten coexpression modules, including 7689 protein-coding genes, were constructed by WGCNA, six of which are considered to be significantly related to the development of the hair follicle cycle. A functional enrichment analysis for each model showed that they are closely related to ECM- receptor interaction, focal adhesion, PI3K-Akt signaling pathway, estrogen signaling pathway, and so on. Combined with the analysis of differential expressed genes, 12 hub genes from coexpression modules were selected as candidate markers, i.e., *COL1A1*, *C1QTNF6*, *COL1A2*, *AQP3*, *KRTAP3-1*, *KRTAP11-1*, *FA2H*, *NDUFS5*, *DERL2*, *MRPL14*, *ANTKMT* and *XAB2*, which might be applied to improve cashmere production.

## 1. Introduction

Hair is one of characteristics of mammals. Hair follicle (HF) is the key organ for hair growth, and it consists of two distinct parts. The upper permanent region is comprised of the infundibulum and isthmus, which are relatively stable, whiles the lower follicle is comprised of the hair bulb and suprabulbar region, which undergo periodic regeneration [1]. Follicular papilla in the hair bulb dictates the induction and maintenance of the follicular epithelial differentiation, and determines anagen duration, hair shaft diameter and length [1,2]. Hair follicles do not enter the self-renewal cycle until they are fully developed after birth, and experience growth (anagen), regression (catagen), and a rest period (telogen), followed hair shedding [3]. The cyclic change is similar in mammals, but the molecule regulatory mechanism may be different due to the different length of cycle and the species-specific morphologies of HF [4].

Cashmere goats are characterized by a double coat including wool and cashmere. Wool and cashmere are produced by two types of HF. Primary hair follicles (PHFs) produce wool while secondary hair follicles (SHFs) produce cashmere [5]. Studies have shown that the SHFs of cashmere goats undergo obvious periodic changes [6,7]. The structural characteristics of the SHFs directly affect the output and quality of cashmere. Cashmere is a luxurious material which is of great economic value in the textile industry [8]. It is, therefore, important to research the mechanism of the periodic development of HFs in order to improve cashmere performance through artificially adjusting the HF cycle.

With the rapid development of high-throughput sequencing technology, some regulatory factors and signal pathways involved in the hair follicle cycle (HFC) have been found through differential expression gene (DEG) analysis and functional enrichment analysis. These well-known regulatory molecules and signal pathways include Wnt/β-catenin [9,10,11], bone morphogenetic proteins (BMPs) [12,13], sonic hedgehog (SHH) [14], notch [15], fibroblast growth factors (FGFs) [16], transforming growth factors (TGFs) [17] and keratin-associated proteins (KRTAPs) [18], etc. Most of them were reported in mice and human, but for cashmere goat with a double-coat some other uncommon regulatory molecules, different signal pathways may be involved. Various researches and analysis methods have made it be possible to determine some unexpected links among the genes expressed in these pathways.

Based on the coat complexity of cashmere goat, it is necessary to systematically study the interconnectedness among genes. The network analysis is a global approach, in contrast to focusing on individual genes. Compared with publicly available cluster analysis statistics software like self-organizing map, hierarchical clustering and k-means [19], weighted gene coexpression network analysis (WGCNA) employs soft thresholding techniques, which results in weighted networks and yields highly robust biological results [20]. Moreover, WGCNA can make full use of the expression of all genes, combine Gene Ontology (GO) enrichment analysis, topology enrichment, KEGG enrichment analysis and gene regulatory network analysis, and identify hub genes [21,22,23]. WGCNA has shown its potential to unravel the gene regulatory architecture of complex traits [24,25,26,27] by clustering genes which are strongly correlated with phenotype or sample characteristics into a module [28].

Herein, we used WGCNA technology to explore the gene coexpression network and identify hub genes closely related to each stage of the HFC of cashmere goats, according to the correlation between genes and hair follicle development characteristics. Our results will provide some other pathways for further research of the regulation mechanism of the hair follicle regeneration cycle in cashmere goats.

## 2. Materials and Methods

### 2.1. Data Sources

Raw data (SRP145408) in this study were downloaded from the Sequence Read Archive database of the NCBI (https://www.ncbi.nlm.nih.gov/) [18]. This dataset contained transcriptome sequencing information from skin tissues of three adult Inner Mongolia cashmere goats collected continuously once a month for 12 months. A total of 36 samples were used.

### 2.2. Data Preprocessing

The raw data were converted into FASTQ format by SRAtools (Version 2.8.1) software [29] and quality control was detected by FastQC (Version 0.11.9) (https://www.bioinformatics.babraham.ac.uk/projects/fastqc/). After filtered adaptor sequences and low-quality reads by Trimmomatic (Version 0.39) software [30], clean reads were mapped to the goat reference genome (ftp://ftp.ensembl.org/pub/release-101/fasta/capra_hircus/dna/) using HISAT2 (version 2.2.0) [31,32]. Counts of the mapped reads were extracted by FeatureCounts [33] and the value of FPKM (Fragments per Kilobase of transcript per million) was obtained using StringTie (Version 2.1.2) [34] to normalize the mapped reads and construct the gene expression matrix for WGCNA analysis.

### 2.3. The Construction of Weighted Gene Coexpression Network for HFC Development

A weighted coexpression network was performed using WGCNA (version 1.69) [21], which was built in R 4.0.2 using RStudio (http://www.rstudio.org), an integrated development environment for R. First of all, the expression matrix was standardized by log2(FPKM+1), and genes with expression standard deviations (SDs) of less than 0.5 were removed in each sample. Based on the correlations among samples, a clustering dendrogram was drawn to remove outliers. Then, a correlation matrix was constructed using pairwise Pearson Correlations among all genes. To achieve a scale-free network, an appropriate soft threshold power β was employed to calculate the adjacency between genes by the pickSoftThreshold function. The power β is a weighted parameter to highlight the strong correlation between genes. Finally, to identify gene modules, the topological overlap measure (TOM) was used to calculate the degree of correlation [20], and a hierarchical clustering tree was constructed according to the corresponding dissimilarity (1-TOM) with the minModuleSize 30 [35]. The genes with similar expression patterns were summarized in the same module by the module eigengenes (MEs) [36]. The modules with more than 75% similarity were merged by using the default tree height cut of 0.25: MEDISSTHRES=0.25 in WGCNA [36,37].

### 2.4. Screening Key Modules Related to HFC

According to the characteristics of the growth and development of HFs in cashmere goat over 12 months [18], we divided the development of HFs into four stages: anagen (April–September), catagen (October and November), telogen (December–February), last-telogen (March). In this study, the key modules related to the periodic regeneration of cashmere goat hair follicle were determined by calculating gene significance (GS) and module membership (MM) values simultaneously [38]. The GS was used to describe the correlation between MEs and HFC trait. The MM indicated the correlation between the gene expression profile and each ME [39]; the higher the correlation in the GS and MM, the more important the module which was associated with the trait [20]. The statistical significance of the correlation between the module and HFC was verified by the Pearson correlation [40]. The modules with |*cor*| > 0.50 (0.50 regarded as a moderate relationship) and *p* < 1 × 10^−4^ (the smaller the *p* value, the more significant the correlation was) were selected as key modules for further analysis [41].

### 2.5. Enrichment Analysis of Genes in Key Modules

The online tool g:profiler [42] (https://biit.cs.ut.ee/gprofiler/) was used to transform the module gene IDs and GO function annotation with default parameters. There were three sub-ontologies of GO annotation including biological process (BP), cellular component (CC) and molecular function (MF). GO terms with significant enrichment were selected according to *adj-p*-value < 0.05. REVIGO (http://revigo.irb.hr/) was used to remove redundant GO terms. Kyoto Encyclopedia of Genes and Genomes (KEGG) pathway analysis was implemented by KOBAS 3.0 (http://kobas.cbi.pku.edu.cn/kobas3/?t=1) with default parameters, and adj-*p*-value < 0.05 was set as the screening condition for significant enrichment.

### 2.6. Identification of Hub Genes Related to HFC

In this study, hub genes were determined by two methods. One was to screen out hub genes based on GS and MM values. Genes with highest MM and highest GS in modules of interest were considered as candidates. Thus, the intramodular hub genes were chosen based |GS| > 0.2, |MM| > 0.9 with weighted *p* value (*p*.weighted) < 0.01 [43]. The *p*.weighted was calculated by networkScreening function to check the significance of GS and MM, respectively. The other method was Maximal Que Centrality (MCC) algorithm [44] from plug-in cytoHubba in Cytoscape (Version 3.6.0) software [45]. The top ten genes ranked by MCC values were considered as hub candidates [46,47,48,49].

### 2.7. Verification of Hub Genes Combined DEGs Analysis

To determine the differences between expression of the hub genes in different periods, we firstly filtered and removed the genes from the count matrix with low coverage across all samples based on an average CPM value < 1. Then, DEGs were screened from a particular period relative to other periods by the edgeR [50] in R package. The threshold of DEGs was set as |log2 fold change| > 1 and FDR < 0.01. Then, Venny 2.1.0 (https://bioinfogp.cnb.csic.es/tools/venny/) was used to overlap DEGs and hub candidates.

### 2.8. Visualization of the Relationship Network between Hub Genes and Their Target Genes

Based on the coexpression network of MEs, the subnetwork of differentially expressed hub genes interacting with their target genes were extracted and visualized using Gephi (version 0.9.2) [51,52]. Gephi “Modularity” function was used to classify individual nodes from the same identified module by WGCNA into communities to further interrogate the networks [53,54].

## 3. Results

### 3.1. Profiles of Transcriptome Data

**Figure 1:**
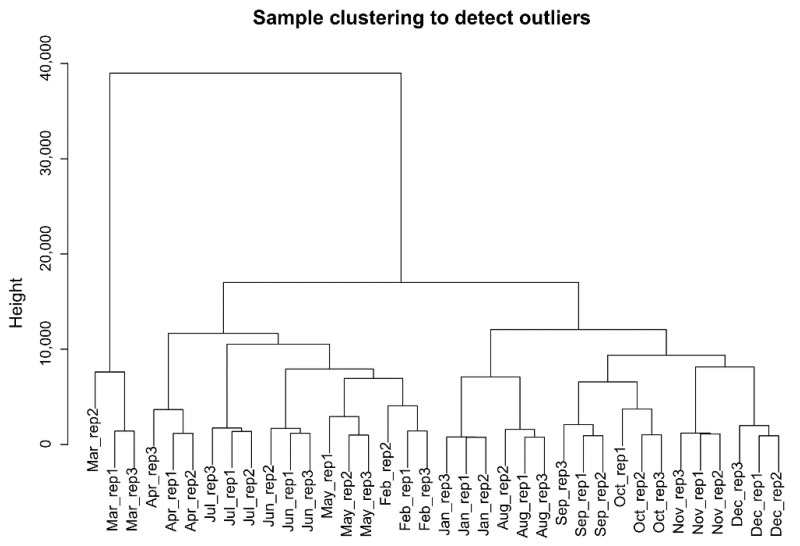
Hierarchical clustering information of samples.

Raw transcriptome data from 36 skin samples were filtered by removing adapter and low-quality fragments to obtain clean reads. The rates of these reads mapping to the reference genome were over 96% (Supplementary Materials Table S1). After normalizing and annotating the mapped reads, a total of 14,240 genes were found. A principal component analysis for all samples indicated that it had a good biological repetition, and the quality of selected data was reliable (Supplementary Materials Figure S1). A hierarchical clustering tree for all samples was drawn to identify the outliers in the samples; the results showed that there was no outlier in the samples, and that all samples could be used in our analysis (Figure 1). After removing the genes with SD < 0.5 between samples, we finally retained 7689 eligible protein-coding genes for subsequent analysis.

### 3.2. Construction of the Weighted Gene Coexpression Network

**Figure 2:**
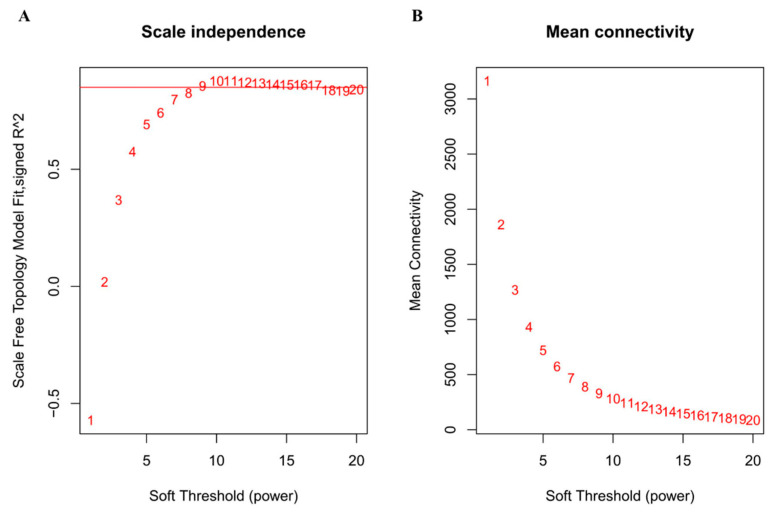
The determination of soft thresholding power. (**A**) is a scale-free fitting index (y-axis) responding to various soft-thresholding powers (x-axis). (**B**) represents the mean connectivity (y-axis) of different soft-thresholding power (x-axis). The approximate scale-free topology can be obtained at the soft-thresholding power of 9.

**Figure 3:**
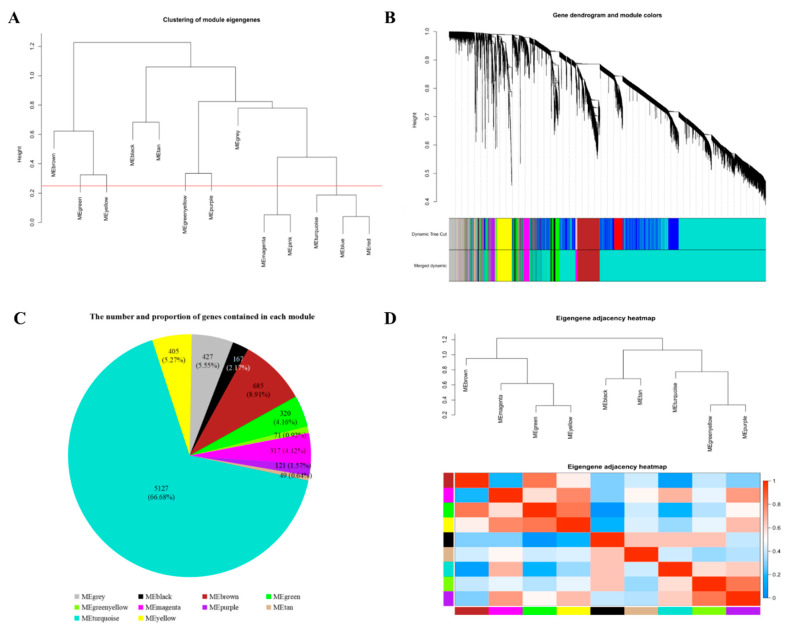
Identification of coexpression modules by weighted gene coexpression network analysis. (**A**) The cluster dendrogram of gene modules eigengenes. Modules with similar expression patterns need to be merged and the red line is the merging threshold. (**B**) The gene clustering dendrogram was obtained according to hierarchic clustering of adjacency-based dissimilarity. The color blocks below the tree graph represent coexpression modules recognized by the dynamic hybrid-cutting method. The genes in grey module were not classified into any coexpression modules. (**C**) The number and proportion of genes contained in each module. (**D**) The adjacency heatmap of eigengene. Above is the module clustering tree, and below is the corresponding module clustering heatmap. Red represents strong correlation and blue represents weak correlation.

To obtain a coexpression network, we used different soft-thresholding power β values, i.e., from 1 to 20, to calculate network structures. When β was set at 9, the scale-free network fitting index reached 0.85 (Figure 2A) and the connectivity between genes in the network was relatively high, which met the scale-free network distribution (Figure 2B). Then, the dynamic hybrid-cutting method was used to cluster genes into modules, and similar modules were merged by setting the MEDissThres cutting line to 0.25 (i.e., the models with 75% of eigengenes similarity were merged) (Figure 3A). ME_pink_ were merged in ME_magenta_, in addition, ME_blue_ and ME_red_ were merged in ME_turquoise_. Finally, ten modules were obtained (Figure 3B). The number and proportion of genes corresponding to the ten modules were calculated and are visualized in Figure 3C. Through the adjacency heatmap of the relationship for each model (Figure 3D), we found that the coexpression relationship of genes from the same module was strong and the ten modules were relatively independent from each other. All corresponding genes in each module can be viewed in Supplementary Materials Table S2.

### 3.3. Key Modules Related to Periodic Development of HFs

**Figure 4:**
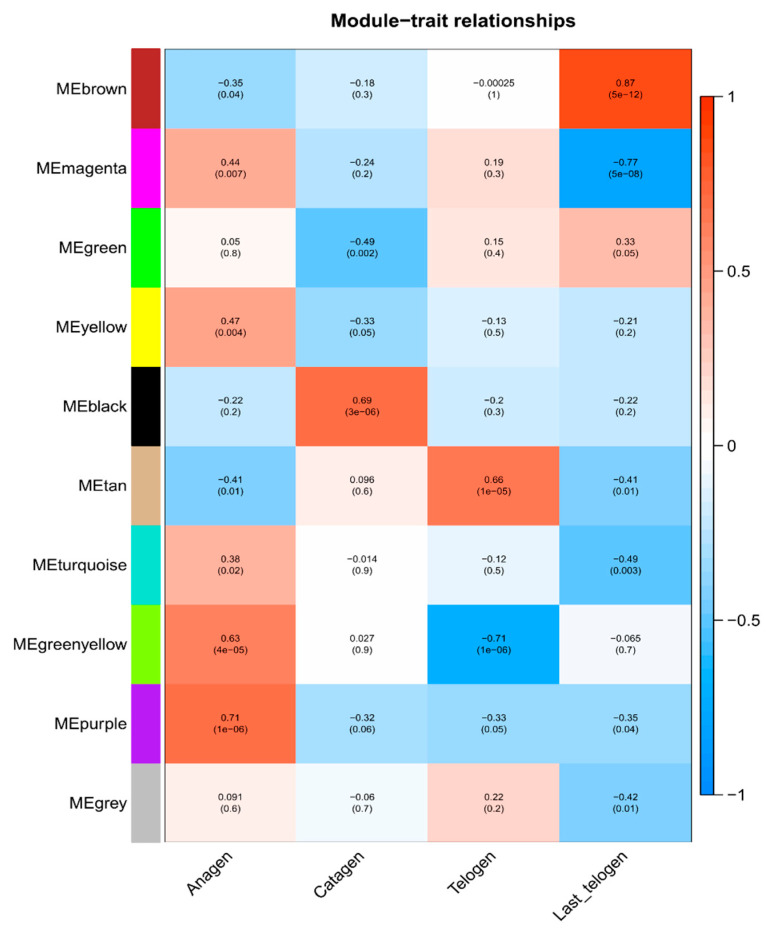
Heatmap of the correlation between module and hair follicle cycle features. The Numbers on the top of the block represent the correlation, the *p* values on the bottom. Red means positive correlation, blue means negative correlation.

To identify these modules related to HFC, the module-trait correlation (Figure 4) and GS values in four stages (Supplementary Materials Figure S2) were calculated. Six out of ten modules were identified to be closely related to the HFC in cashmere goats. The ME_greenyellow_ (*cor* = 0.63, *p* = 4 × 10^−5^) and ME_purple_ (*cor* = 0.71, *p* = 1 × 10^−6^) were positively correlated with HFs development in anagen (Figure 4 and Supplementary Materials Figure S2A). ME_black_ was positively related with HFs development in catagen (*cor* = 0.69, *p* = 3 × 10^−6^) (Figure 4 and Supplementary Materials Figure S2B). ME_tan_ was positively related with HFs development in telogen (*cor* = 0.66, *p* = 1 × 10^−5^), while ME_greenyellow_ was negatively correlation in telogen (*cor* = −0.71, *p* = 1 × 10^−6^) (Figure 4 and Supplementary Materials Figure S2C). ME_brown_ was positively related with HFs development in the late-telogen (*cor* = 0.87, *p* = 5 × 10^−12^), but ME_magenta_ was negatively correlation in late-telogen (*cor* = −0.77, *p* = 5 × 10^−8^) (Figure 4 and Supplementary Materials Figure S2D).

### 3.4. Gene Enrichment Analysis from Key Modules

To explore the molecular functions and biological pathways of genes in key modules significantly associated with the periodic development of the HF, we performed GO enrichment analysis for the six module genes mentioned above. The genes of ME_greenyellow_ and ME_black_ were mainly involved in cytoskeleton, supramolecular complex and intermediate filament. The ME_tan_ genes were related to fatty acid biosynthetic process and response to corticotropin-releasing hormone. The ME_purple_ genes were involved in extracellular matrix, extracellular region and collagen trimer. The ME_brown_ genes were enriched in intracellular, organelle, ribosome and mitochondrion, and participated in metabolic process. The ME_magenta_ genes were enriched in the extracellular and membrane, and related to regulation of response to stimulus (Supplementary Materials Figure S3 and Supplementary Materials Table S3).

KEGG pathway analysis showed that the ME_greenyellow_ and ME_purple_ genes related to anagen period were enriched in complement and coagulation cascades and PI3K-Akt signaling pathway, focal adhesion and extracellular matrixc (ECM)-receptor interaction (Table 1), respectively. The significant pathway in ME_black_ related to Catagen was estrogen signaling pathway. The genes of ME_tan_ and ME_greenyellow_ related to telogen period were enriched in glycerolipid metabolism, fat digestion and absorption, and complement and coagulation cascades, respectively. The genes of ME_brown_ and ME_magenta_ related to last-telogen were focused on ribosome and infection (Table 1), respectively.

### 3.5. Hub Genes Closely Related to Different Stages of Hair Follicle Development

We used two methods to identify hub genes correlated with the periodic development of HFs. Based on MM and GS values of the genes in each of the key modules to select the hub genes, |GS| > 0.2, |MM| > 0.9 and *p*.weighted < 0.01 were used as the identification criteria. As a result, 11, 14, 20, 8, 260 and 21 genes were obtained from ME_greenyellow_, ME_purple_, ME_black_, ME_tan_, ME_brown_ and ME_magenta_, respectively (Supplementary Materials Table S4). In addition, MCC algorithm in cytoHubba plugin from Cytoscape software were used to screen hub candidates (Supplementary Materials Table S5) [48]. Combining these two methods, finally, a total of 38 hub genes were found: six in ME_greenyellow_, seven in ME_purple_, eight in ME_black_, six in ME_tan_, ten in ME_brown_ and one in ME_magenta_ (Supplementary Materials Figure S4).

### 3.6. Verification of Hub Genes by DEGs Analysis

**Figure 5:**
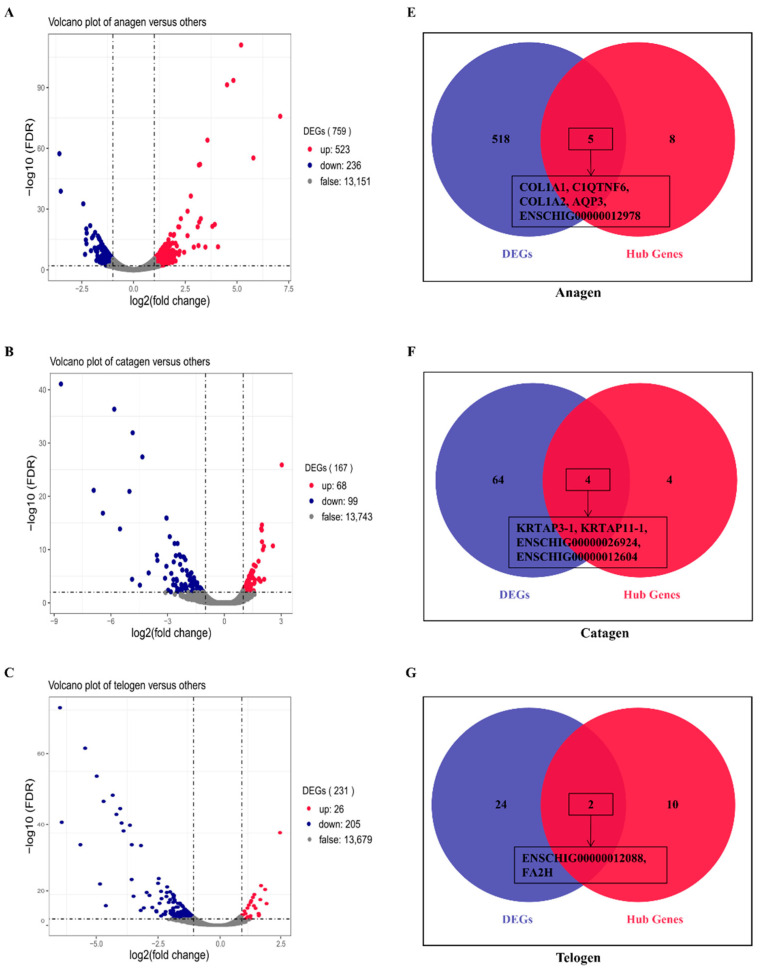

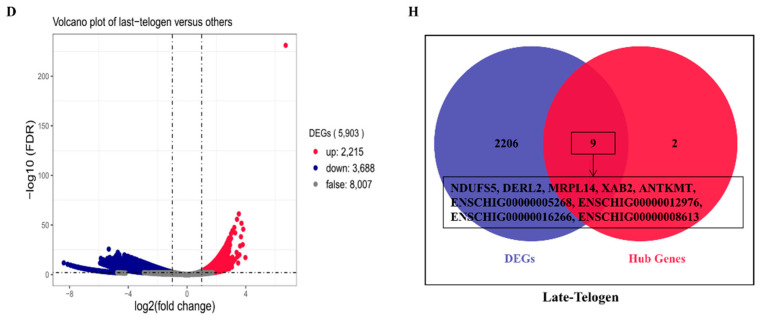
Differential expression hub genes related to different stages of hair follicle regeneration. The results of differential expression genes analysis in (**A**) anagen (**B)** catagen, (**C**) telogen and (**D**) late-telogen compared with nonself stages. Overlapping results of up-regulated differential expression genes and hub genes in (**E**) anagen, (**F**) catagen, (**G**) telogen and (**H**) late-telogen.

**Figure 6:**
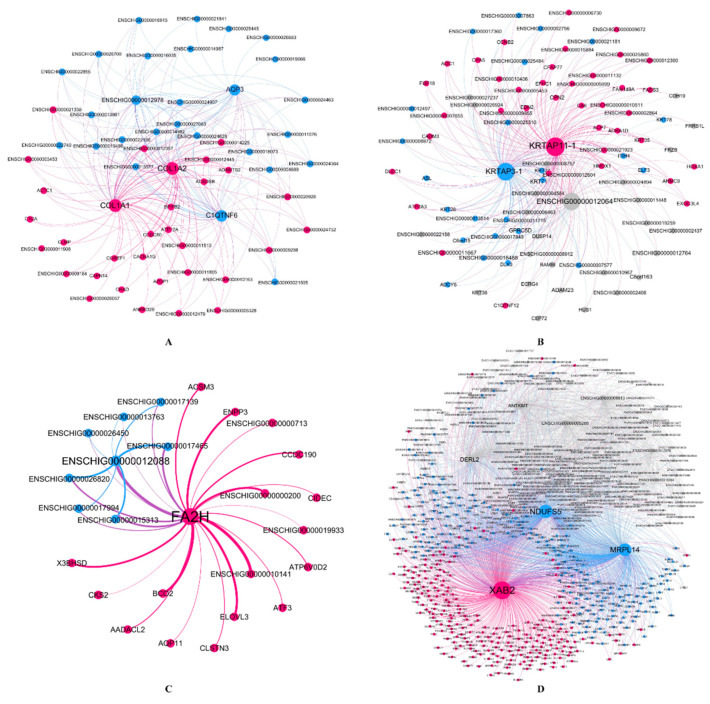
Visualization of coexpression network between hub genes with interaction genes in four stages. (**A**) anagen, (**B**) catagen, (**C**) telogen and (**D**) late-telogen. The size of the circle represents the degree values of the adjacency genes in the network from WGCNA. Nodes and edges were colored based on Gephi modularity class to classify individual nodes into network communities. The thickness of the edge represents the strength of the interaction between genes, and the thicker the line, the stronger the interaction.

**Figure 7:**
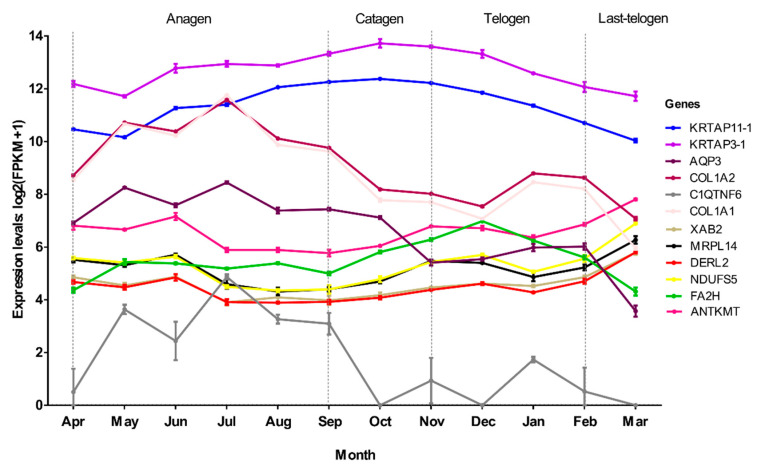
Line chart of the expression changes of 12 hub genes throughout the hair follicle cycle of cashmere goats.

To verify the relationship between hub genes and HFs development, we detected the expression of hub genes by DEGs analysis based on the filtered 13,910 genes at a given period compared with other periods. The DEGs analysis results were shown in Figure 5A–D. The numbers of DEGs in anagen vs. others, catagen vs. others, telogen vs. others and last-telogen vs. others were respectively 759 (up-regulated 523, down-regulated 236), 167 (up-regulated 68, down-regulated 99), 231 (up-regulated 26, down-regulated 205) and 5903 (up-regulated 2215, down-regulated 3688). Using the online software Venny to overlap hub genes and DEGs, it showed that 20 genes out of 38 hub genes were overlapped to up-regulated DEGs including five hub genes in anagen (Figure 5E), four hub genes in catagen (Figure 5F), two hub genes in telogen (Figure 5G) and nine hub genes in last-telogen (Figure 5H). Six genes out of 38 hub genes were overlapped to down-regulated DEGs including five hub genes (*ENSCHIG00000011548*, *ENSCHIG00000014533*, *ENSCHIG00000015017*, *ENSCHIG00000010344* and *ENSCHIG00000000533*) in telogen and one hub gene (ENSCHIG00000009953) in last-telogen. In four stages, the coexpression network of hub genes interacting with multiple genes were visualized (Figure 6A–D), which suggested the importance of these hub genes in the regulation. Twelve genes in all differential expression hub genes have been annotated, including four genes (*COL1A1*, *C1QTNF6*, *COL1A2* and *AQP3*) in anagen, two genes *(KRTAP3-1* and *KRTAP11-1*) in catagen, one gene (*FA2H*) in telogen and five genes (*NDUFS5*, *DERL2*, *MRPL14*, *ANTKMT* and *XAB2*) in last-telogen. These differential hub genes involved in the process and pathways related to cyclic development of hair follicle by coexpression regulation in different modules. To understand the dynamic expression of these 12 annotated genes in the HFC of cashmere goats, their expression levels were visualized in Figure 7.

## 4. Discussion

Hair follicles of cashmere goat undergo annual cyclic changes. The economic value and reference value for research on human hair loss have led researchers to focus on the molecular mechanism of HF regeneration in cashmere goats. Though some genes and signals have been found, precise knowledge is still lacking, due to the polygenes and multiple pathways involved. In this study, WGCNA was used to find hub genes and pathways in different periodical development phases of HFs based RNA-seq data from skins tissues of Inner Mongolia cashmere goats.

Sample hierarchical cluster analysis were performed by WGCNA based on all gene expression levels over 12 months. The results showed that there were three obvious clusters: Mar., Aug to Jan, and Feb to Jul except for Mar (Figure 1). According to the growth cycle of HF, it mainly included three stages: anagen, catagen, and telogen. It seemed that the categories could correspond to the stages, but we found that the gene expression changes of Jan. and Aug., Feb. and May were clustered together (Figure 1), respectively. In fact, for the Inner Mongolia cashmere goat, the cashmere shed in Apr. when it was just the month of collecting cashmere and hair follicle started to grow again until to Sept.

Thus, combined the morphological changes of hair follicle [18], Jan. and Aug., Feb. and May should not be in same stages, respectively. However, the periodical development of hair follicle was induced by the cyclical fluctuation of gene expression, and their expression patterns between different months (Feb. and May, Jan. and Aug.) may be similar and clustered together. For Mar., it was a transition stage from the telogen to the anagen and it was furthest from other months based on the cluster analysis, so it was specified as last-telogen. Finally, we divided the development of HFs into four stages: anagen (April–September), catagen (October and November), telogen (December–February), last-telogen (March).

After constructing the weighted gene coexpression network modules and correlating with the HF development stages, six key modules were found. ME_purple_ and ME_greenyellow_ in anagen focused on ECM-receptor interaction, PI3K-Akt signaling pathway, complement and coagulation cascades (Table 1). Genes from ME_black_ in catagen were associated with epithelial cell differentiation and estrogen signaling pathway (Supplementary Materials Figure S3, Table 1). Genes from ME_tan_ and ME_greenyellow_ in telogen were related to the fatty metabolism and the composition of supramolecular fiber, respectively (Supplementary Materials Figure S3, Table 1). Genes from metabolism-related ME_brown_ and stimulus-related ME_magenta_ the immune response had the higher correlation with late-telogen (Supplementary Materials Figure S3, Table 1). In these models, 12 annotated hub genes were found by coexpression regulation involving in the periodic development of hair follicle (Figure 6).

In anagen, *COL1A1*, *C1QTNF6*, *COL1A2* and *AQP3* were identified as hub genes. *COL1A1* and *COL1A2* encode the proalpha1 and two chains of type I collagen which is the major protein of the extracellular matrix (ECM) [55,56,57]. ECM widely exists in dermal sheath and dermal papilla (DP) in anagen [58,59,60], diminishes during catagen, and is minimal in the telogen follicle [61]. The interaction between DP and ECM plays an important role in HF development. The ECM type I collagen can enhance DP cell aggregation through its effect on cell adhesion and motility, which is essential in hair regeneration [62,63]. In our study, we found that *COL1A1* and *COL1A2* were highly expressed in anagen especially July when the follicle was in vigorous (Figure 7). Moreover, *COL1A1* and *COL1A2* participated not only in ECM-receptor interaction, but also in focal adhesion and PI3K-Akt signaling pathway (Table 1). Focal adhesions can engage with the surrounding ECM [64] and relate to the migration of HF stem cells towards the bulb region [65]. The effect of the PI3K/AKT signaling pathway on the regeneration of hair follicles has also been reported [66]. All these results prove that *COL1A1* and *COL1A2* are essential for regeneration of cashmere hair and can be used as important candidate genes for in-depth study of its function. *AQP3* is an aquaporin coding gene, which is highly expressed in epidermal keratinocytes [67,68,69]. It plays a decisive role in epidermal proliferation and skin damage repair by mediating the transport of water and glycerol [6,70]. *AQP3* can participate in the migration and proliferation of keratinocytes [70], and provide glycerol for phospholipase D2 (PLD2) to synthesize phosphatidylglycerol (PG), negative feedback inhibits keratinocyte proliferation and promote keratinocyte differentiation [71,72]. Our research showed that *AQP3* was highly expressed in anagen (Figure 7) and interacted with *COL1A1* and *COL1A2*. Recently, *AQPs* was clustered in the group of seasonal rhythm genes in cashmere goat skin [73], which indicated these genes played an important role in maintain the specific rhythm of the HF growth.

In catagen, *KRTAP3-1* and *KRTAP11-1* were identified as hub genes. *KRTAP3-1* and *KRTAP11-1* belong to keratin-associated proteins (KRTAPs) family. KRTAPs are the main structural protein molecules of hair fibers. A large number of studies have shown that KRTAPs are highly expressed in the cortex area of hair fibers and play a key role in the physical properties of hairs [74,75,76]. The dynamic change of KRTAPs throughout HFC affects the growth of hair follicle and hair shaft [74,77]. It was showed *KRTAP11-1* was mainly expressed in catagen and telogen and could interact with other genes to regulate the HFs development [78]. *KRTAP11-1* not only promoted the expression of catagen-inducing factors (*BMP2* and *TGFβ1*), but also inhibited the expression of growth-activator (*LEF-1*) [78]. It was reported *KRTAP3-1* expressed highly in September than February when only comparing these two months in Shanbei cashmere goat [79]. In this study, we found that both *KRTAP11-1* and *KRTAP3-1* were highly expressed throughout HFC and reached to the peak in October (catagen) (Figure 7). If only comparing September and February, it had similar results for these two genes expression.

The telogen can be divided into early-refractory and last-competent stages. Conventionally, the telogen is merely an idle waiting period for HFs to enter the anagen of proliferation and differentiation. But new study argues that the telogen is a complicated stage in which HFs are biologically active [80]. During refractory telogen stage, HFs are insensitive to response anagen-inducing stimuli [81]. In this study, we only found one hub genes, fatty acid 2-hydroxylase (*FA2H*), was highly expressed in cashmere skin in the telogen. *FA2H* is mainly participates in the biological oxidation of fatty acids (Supplementary Materials Table S3) and the synthesis of 2-hydroxylated sphingolipids, which is believed to play a role in the formation of epidermal barrier [82,83,84,85]. Previous study has shown that *FA2H* in mouse is only expressed in sebaceous glands of skin, but the absence of *FA2H* can delay hair generation with hair loss in telogen period [83]. This suggests that *FA2H* plays important roles in maintaining the hair follicle homeostasis in the early telogen period.

Indeed, the growth traits of HFs in last-telogen are the lowest value in histology throughout HFC [3,7,18], but some people thought last-telogen is the master-switch that controls HFC by retaining hair fibers with minimal energy expenditure and responding to a variety of stimuli to launch a new regeneration cycle [80,86]. In this study, genes in ME_brown_ and ME_magenta_ associated with last-telogen were involved in metabolic process and stimulus response and immunity, respectively (Table 1, Supplementary Materials Tables S3 and S4). In addition, increasing research showed that mitochondria could determine the differentiation and proliferation of hair cell upon injury by regulating energy metabolism [87,88], regulate skin aging and hair loss [89]. Mitochondria metabolic disorders can lead to hair and skin abnormalities [90]. Five hub genes were found in last-telogen, i.e., *NDUFS5*, *DERL2*, *MRPL14*, *ANTKMT* and *XAB2*. Among these, *DERL2* and *XAB24*, *NDUFS5* and *MRLP1* had a similar expression profile across the year (Figure 7). *NDUFS5* and *MRPL14* participated in the synthesis of mitochondrial protein complex and ribosomal subunits. *DERL2, ANTKMT* and *XAB2* were mainly involved in the metabolic process. According to coexpression network, these hub genes interacted with many of other genes to form a large and complex signaling network (Figure 6D). They might be crucial to conserving energy and initiating new cycles during last-telogen. Further research is needed to fully uncover the magic of last-telogen.

## 5. Conclusions

In this study, WGCNA was used to construct a gene-weighted coexpression network to explore key gene modules and hub genes closely related to the development of HFC in cashmere goats. Six key modules were identified, and genes in models were enriched using well-known biological processes or pathways, such as epithelial cell differentiation, ECM-receptor interaction, and the PI3K-Akt signaling pathway. Moreover, we also found metabolism process, response, and immunity related pathways in last-telogen. Twelve hub genes with significant expression changes in different development stages of HFs were selected as candidate marks. All of these findings help us better understand the internal mechanism of hair follicle growth and development in cashmere goats. In addition, these hub genes might be a medium to artificially regulate and control the production of cashmere.

## Figures and Tables

**Table 1:** KEGG pathways in coexpression modules.

Modules	Terms	Adj-*p*-Value	Gene Counts	Genes
ME_greenyellow_	Complement and coagulation cascades	0.004	4	*C1QC*; *C1QB*; *ENSCHIG00000015928*; *ENSCHIG00000021894*
ME_purple_	ECM-receptor interaction	0.002	5	*COL6A2*; *COL1A2*; *THBS3*; *CHAD*; *COL1A1*
	PI3K-Akt signaling pathway	0.009	7	*THEM4*; *CHAD*; *THBS3*; *CREB3L1*; *COL1A2*; *COL1A1*; *COL6A2*
	Focal adhesion	0.016	5	*COL6A2*; *COL1A2*; *THBS3*; *CHAD*; *COL1A1*
	Human papillomavirus infection	0.022	6	*CHAD*; *THBS3*; *CREB3L1*; *COL1A2*; *COL1A1*; *COL6A2*
ME_black_	Staphylococcus aureus infection	5.24 × 10^−9^	10	*KRT36*; *KRT35*; *KRT28*; *KRT27*; *C5AR1*; *ENSCHIG00000011715*; *ENSCHIG00000017849*; *ENSCHIG00000021923*; *ENSCHIG00000022158*; *ENSCHIG00000025510*
	Estrogen signaling pathway	8.36 × 10^−7^	10	*KRT36*; *KRT35*; *KRT28*; *KRT27*; *ADCY5*; *ENSCHIG00000011715*; *ENSCHIG00000012764*; *ENSCHIG00000017849*; *ENSCHIG00000021923*; *ENSCHIG00000025510*
ME_tan_	Glycerolipid metabolism	9.82 × 10^−5^	5	*GLYCTK*; *ENSCHIG00000010141*; *ENSCHIG00000012088*; *ENSCHIG00000015313*; *ENSCHIG00000017465*
	Fat digestion and absorption	0.001	4	*ENSCHIG00000010141*; *ENSCHIG00000012088*; *ENSCHIG00000015313*; *ENSCHIG00000017465*
ME_brown_	Ribosome	2.00 × 10^−7^	22	*RPL36*; *MRPS15*; *MRPL27*; *MRPL24*; *MRPL21*; *MRPL14*; *ENSCHIG00000000917*; *ENSCHIG00000000315*; *ENSCHIG00000003454*; *ENSCHIG00000005169*; *ENSCHIG00000006564*; *ENSCHIG00000007457*; *ENSCHIG00000007926*; *ENSCHIG00000009094*; *ENSCHIG00000010382*; *ENSCHIG00000010444*; *ENSCHIG00000011183*; *ENSCHIG00000011261*; *ENSCHIG00000011972*; *ENSCHIG00000013535*; *ENSCHIG00000019224*; *ENSCHIG00000023286*
	Oxidative phosphorylation	0.004	14	*SDHC*; *NDUFS5*; *NDUFB9*; *NDUFB7*; *NDUFB10*; *NDUFAB1*; *COX7A2L*; *COX17*; *ATP6V0C*; *ENSCHIG00000003884*; *ENSCHIG00000008372*; *ENSCHIG00000008931*; *ENSCHIG00000012976*; *ENSCHIG00000015565*
	Nonalcoholic fatty liver disease (NAFLD)	0.009	14	*SDHC*; *NR1H3*; *NDUFS5*; *NDUFB9*; *NDUFB7*; *NDUFB10*; *NDUFAB1*; *GSK3A*; *COX7A2L*; *CEBPA*; *ENSCHIG00000003884*; *ENSCHIG00000008372*; *ENSCHIG00000012976*; *ENSCHIG00000015565*
ME_magenta_	Antigen processing and presentation	0.001	8	*PDIA3*; *CTSL*; *ENSCHIG00000008848*; *ENSCHIG00000009614*; *ENSCHIG00000012657*; *ENSCHIG00000018922*; *ENSCHIG00000019468*; *ENSCHIG00000024246*
	Epstein-Barr virus infection	0.001	13	*PDIA3*; *NFKBIA*; *ICAM1*; *CD19*; *CCND3*; *AKT1*; *ENSCHIG00000008848*; *ENSCHIG00000009614*; *ENSCHIG00000012657*; *ENSCHIG00000018922*; *ENSCHIG00000019468*; *ENSCHIG00000019897*; *ENSCHIG00000024246*
	Human immunodeficiency virus 1 infection	0.012	12	*TNFRSF1A*; *STING1*; *PDIA3*; *NFKBIA*; *CFL1*; *AKT1*; *ENSCHIG00000008848*; *ENSCHIG00000009614*; *ENSCHIG00000012657*; *ENSCHIG00000018922*; *ENSCHIG00000019468*; *ENSCHIG00000024246*
	Human T-cell leukemia virus 1 infection	0.016	12	*TNFRSF1A*; *NFKBIA*; *ICAM1*; *CCND3*; *AKT1*; *ENSCHIG00000008848*; *ENSCHIG00000009614*; *ENSCHIG00000012562*; *ENSCHIG00000012657*; *ENSCHIG00000018922*; *ENSCHIG00000019468*; *ENSCHIG00000024246*
	Human cytomegalovirus infection	0.021	12	*TNFRSF1A*; *STING1*; *PDIA3*; *NFKBIA*; *AKT1*; *ENSCHIG00000008848*; *ENSCHIG00000009614*; *ENSCHIG00000011782*; *ENSCHIG00000012657*; *ENSCHIG00000018922*; *ENSCHIG00000019468*; *ENSCHIG00000024246*
	Fluid shear stress and atherosclerosis	0.035	9	*TNFRSF1A*; *PECAM1*; *ICAM1*; *CTSL*; *AKT1*; *ACVR2B*; *ENSCHIG00000008471*; *ENSCHIG00000026143*; *ENSCHIG00000026491*

## Data Availability

All data analyzed during this study are available in the website (https://www.ncbi.nlm.nih.gov/sra/?term=SRP145408).

## Supplementary Materials

The following are available online at https://www.mdpi.com/2073-4425/12/2/180/s1, Figure S1: PCA analysis results of 36 samples. Figure S2: The histogram of average gene significance (GS) in the modules associated with the different stages of the hair follicle cycle. (A–D) represented the GS values in anagen, catagen, telogen and last-telogen period, respectively. Figure S3: GO enrichment analysis results of each module genes. Figure S4: Screening of key genes in the network. The blue represents hub genes exported by CytoHubba, the red represents hub genes obtained by |GS| > 0.2 and |MM| > 0.9 criterion, and the intersection represents the common hub genes screened by both methods. Table S1: Alignment rates of effective RNA-seq sequences on the reference genome. Table S2: The list of genes contained in each module. Table S3: Go functional annotation of genes from the modules significantly related to the cyclic development of hair follicle. Table S4: The hub candidates table of GS and MM values form WGCNA. Table S5: The top ten hub candidates with MCC values form CytoHubba.
