# Supplementary material for: Identification of Key Pathways and Genes Related to the Development of Hair Follicle Cycle in Cashmere Goats
Source: Genes (Basel). 2021 Jan 27;12(2):180. doi: 10.3390/genes12020180 (PMC7911279; doi:10.3390/genes12020180)
Supplement: Supplementary file 1 [file genes-12-00180-s001.zip › Supplemental Figure S2.pdf]

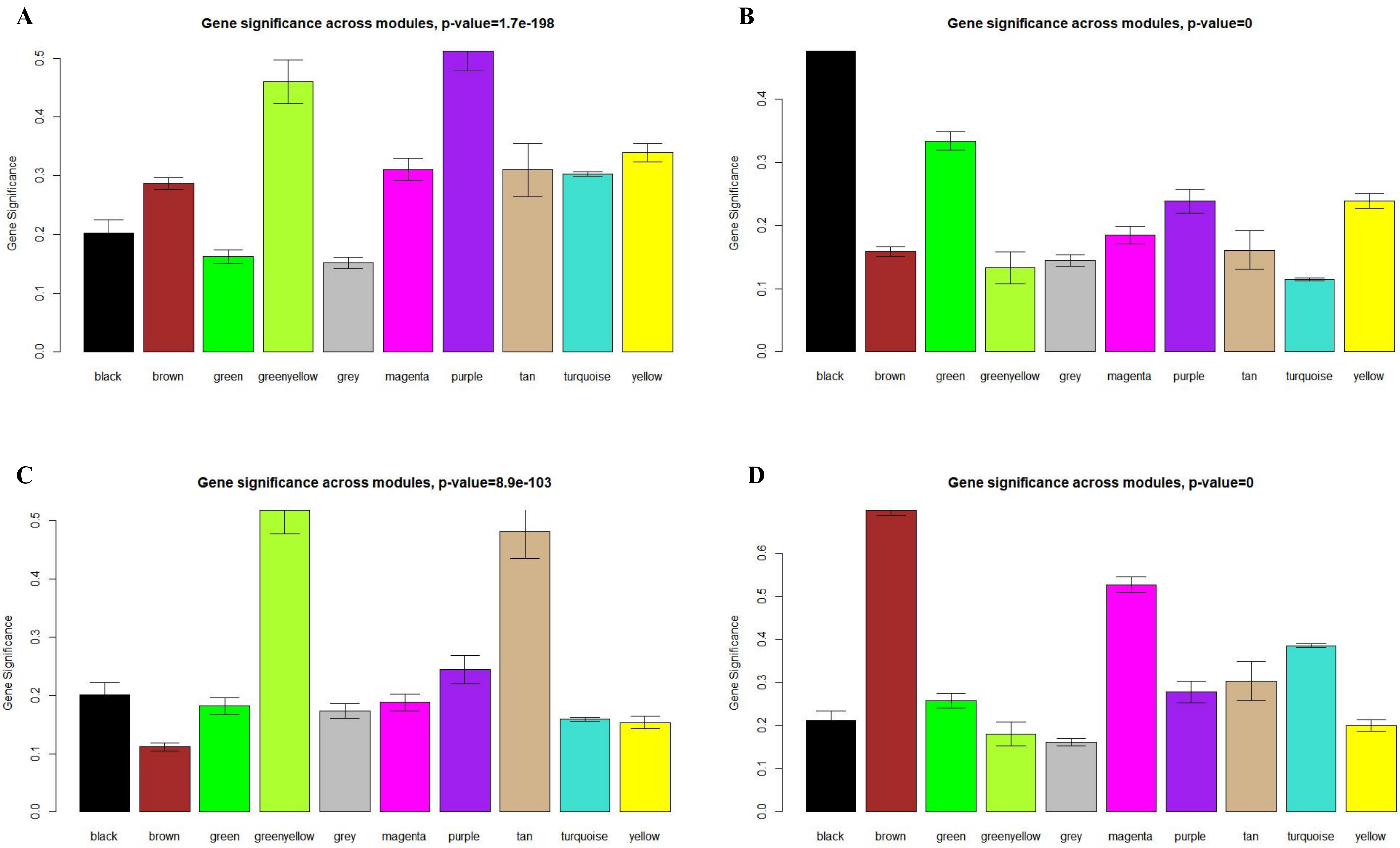
A
Gene significance across modules, p-value=1.7e-198
Gene Significance
0.0
0.1
0.2
0.3
0.4
0.5
black
brown
green
greenyellow
grey
magenta
purple
tan
turquoise
yellow
B
Gene significance across modules, p-value=0
Gene Significance
0.0
0.1
0.2
0.3
0.4
black
brown
green
greenyellow
grey
magenta
purple
tan
turquoise
yellow
C
Gene significance across modules, p-value=8.9e-103
Gene Significance
0.0
0.1
0.2
0.3
0.4
0.5
black
brown
green
greenyellow
grey
magenta
purple
tan
turquoise
yellow
D
Gene significance across modules, p-value=0
Gene Significance
0.0
0.1
0.2
0.3
0.4
0.5
0.6
black
brown
green
greenyellow
grey
magenta
purple
tan
turquoise
yellow
