# Supplementary figures and images for: Identification of Key Pathways and Genes Related to the Development of Hair Follicle Cycle in Cashmere Goats
Source: Genes (Basel). 2021 Jan 27;12(2):180. doi: 10.3390/genes12020180 (PMC7911279; doi:10.3390/genes12020180)

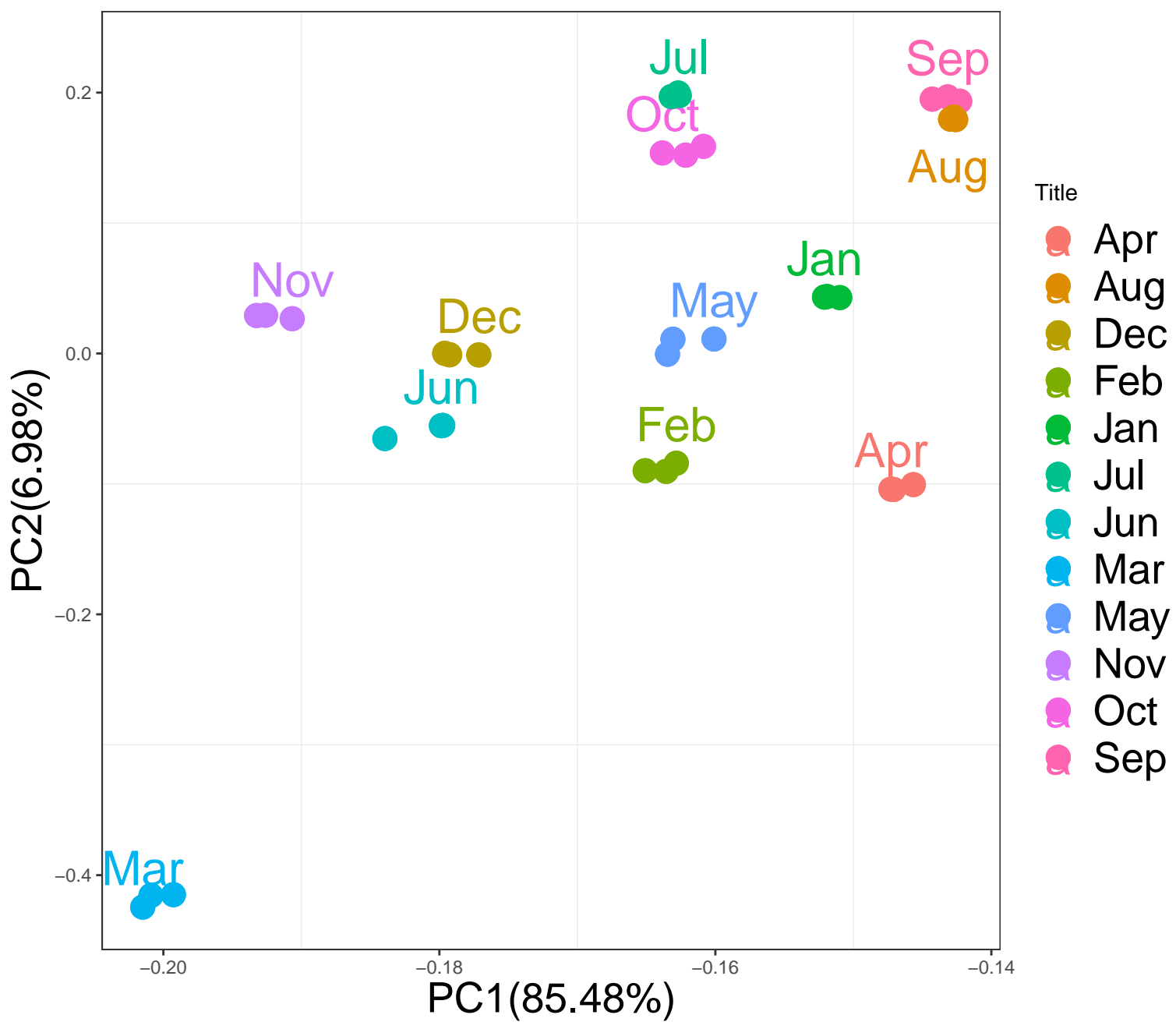

Supplement: Supplementary file 1 [file genes-12-00180-s001.zip › Supplemental Figure S1.pdf]

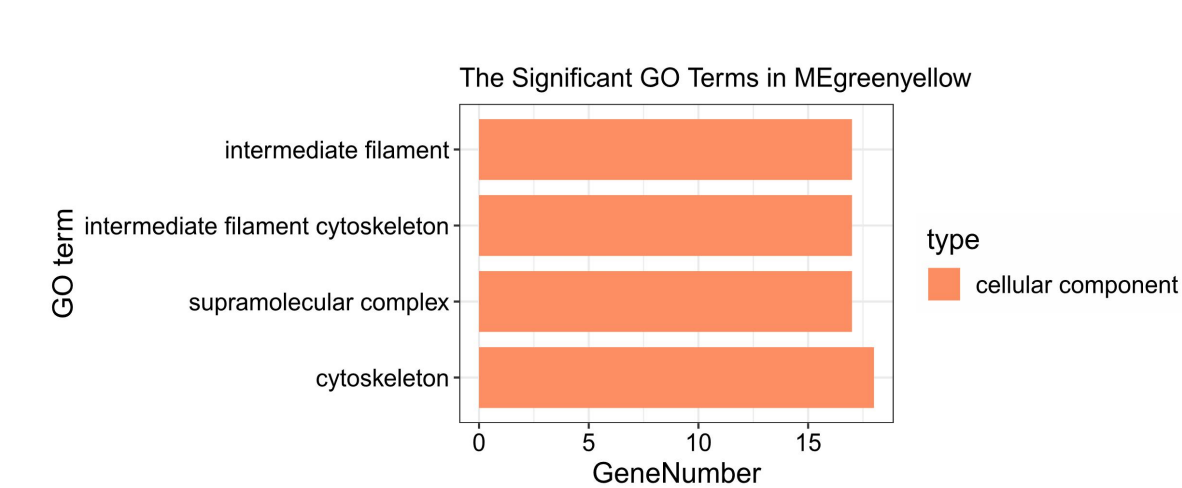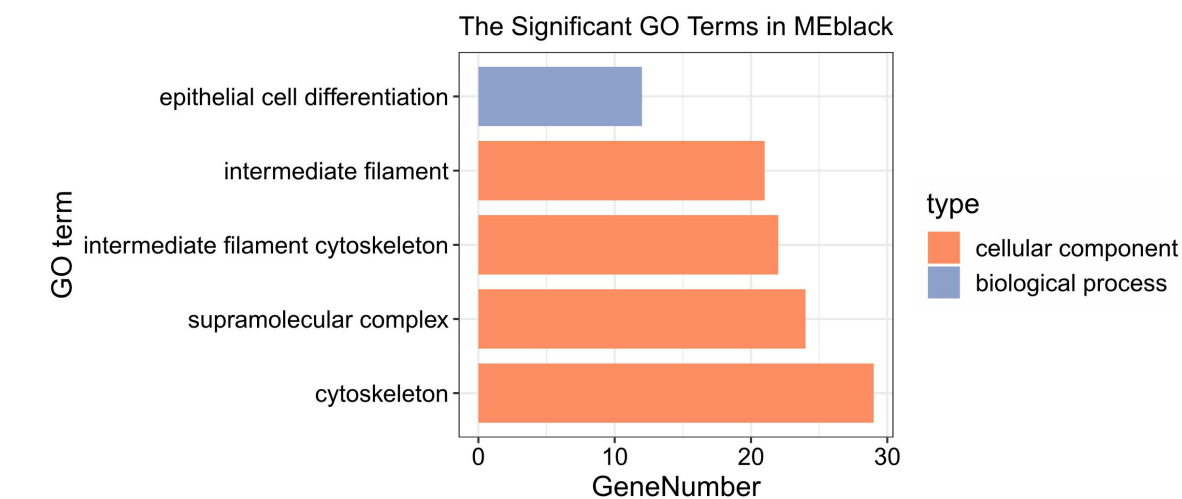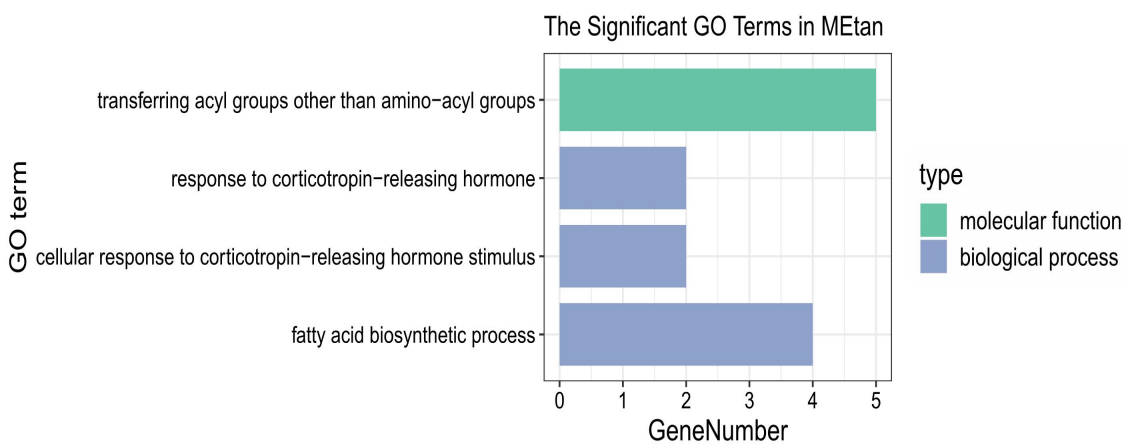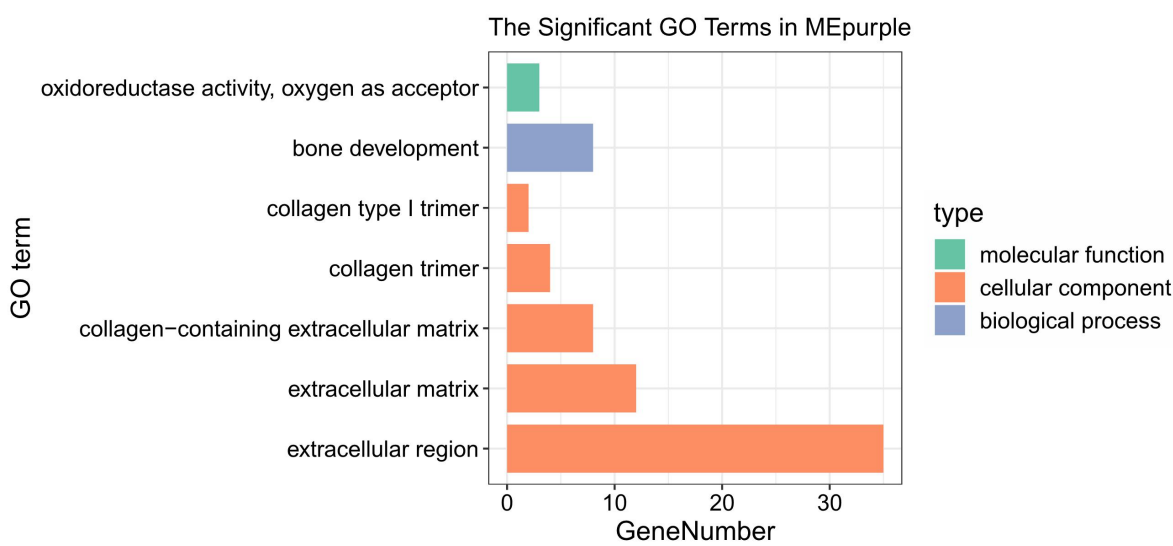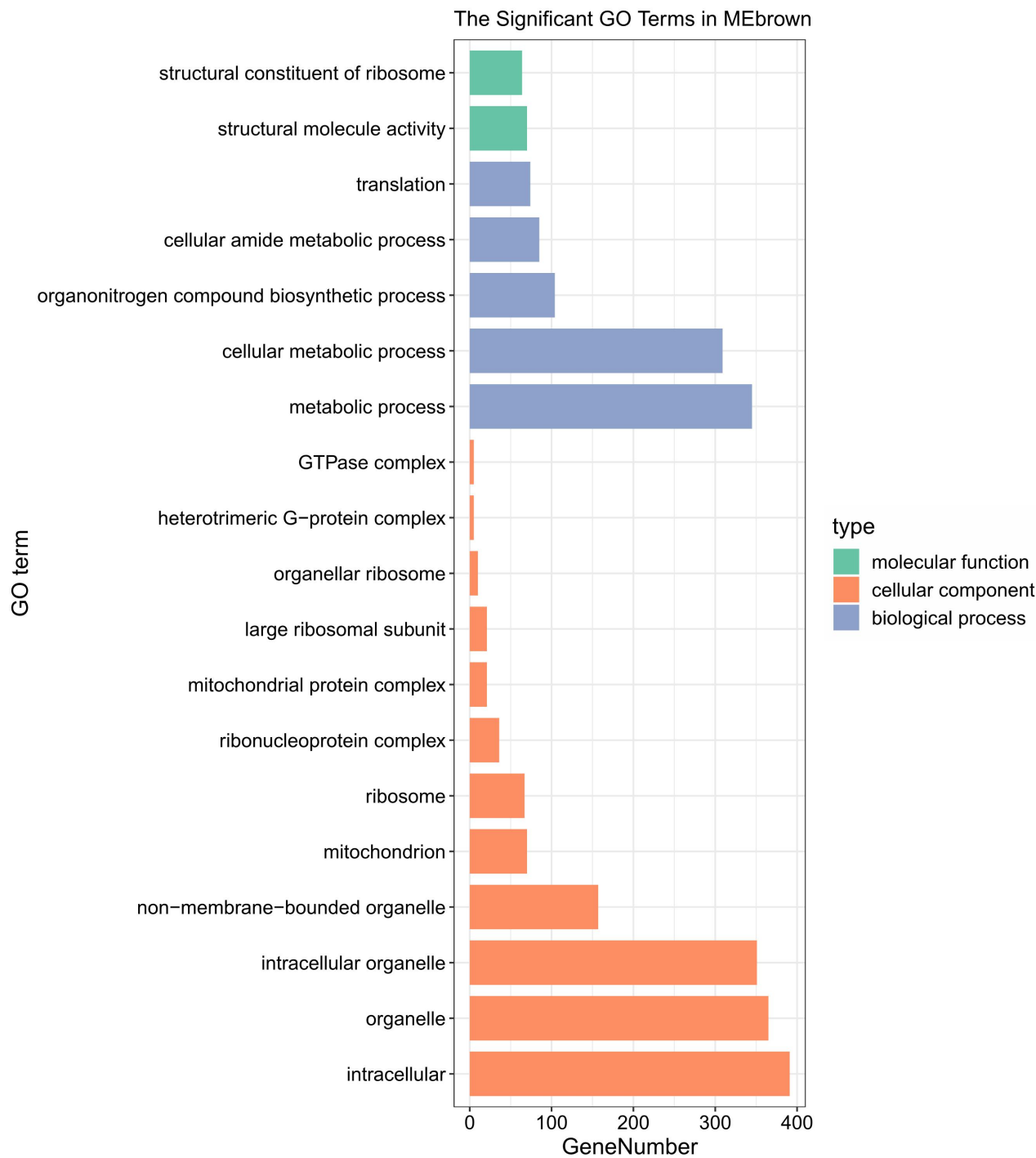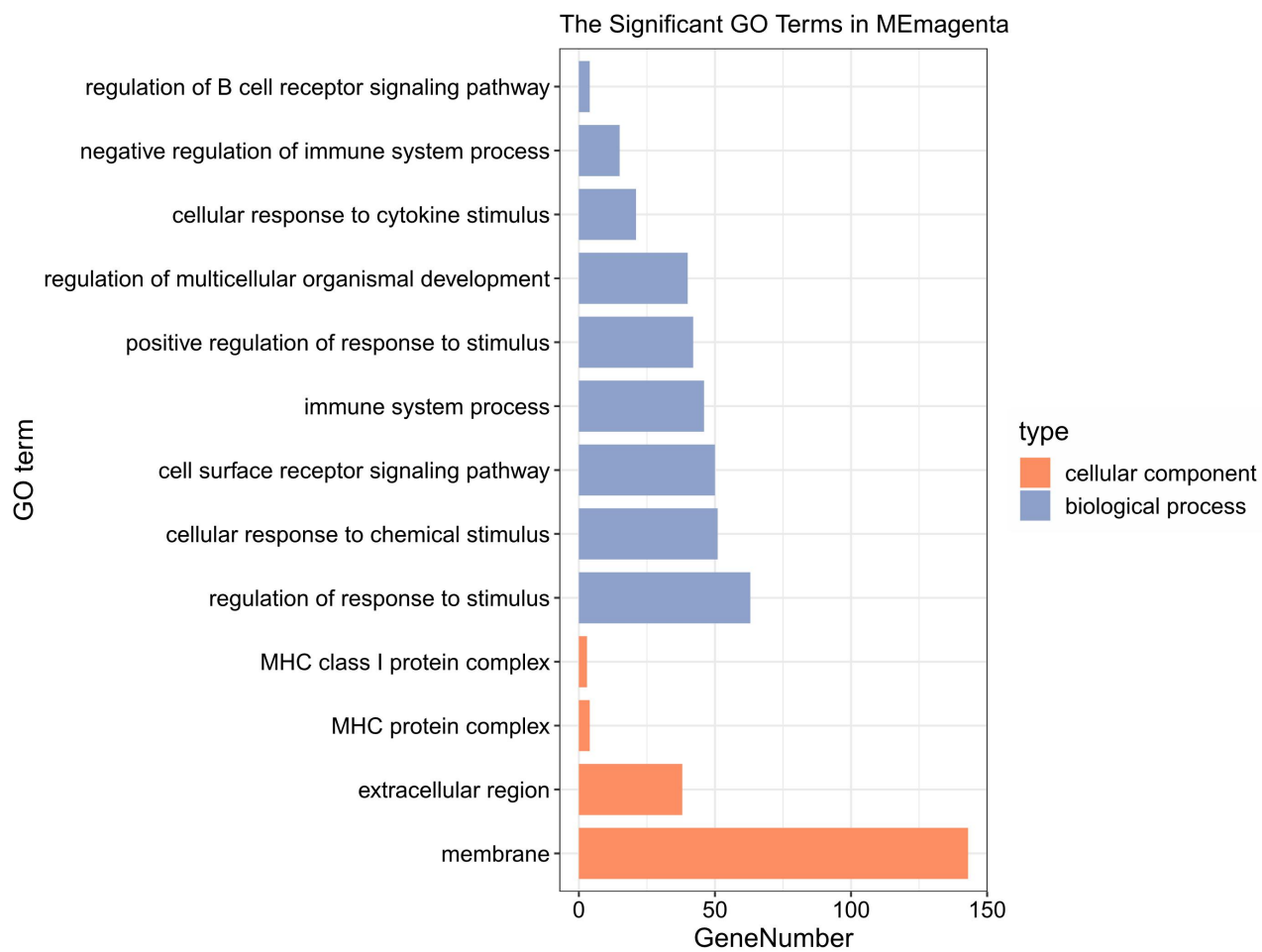

Supplement: Supplementary file 1 [file genes-12-00180-s001.zip › Supplemental Figure S3.pdf]

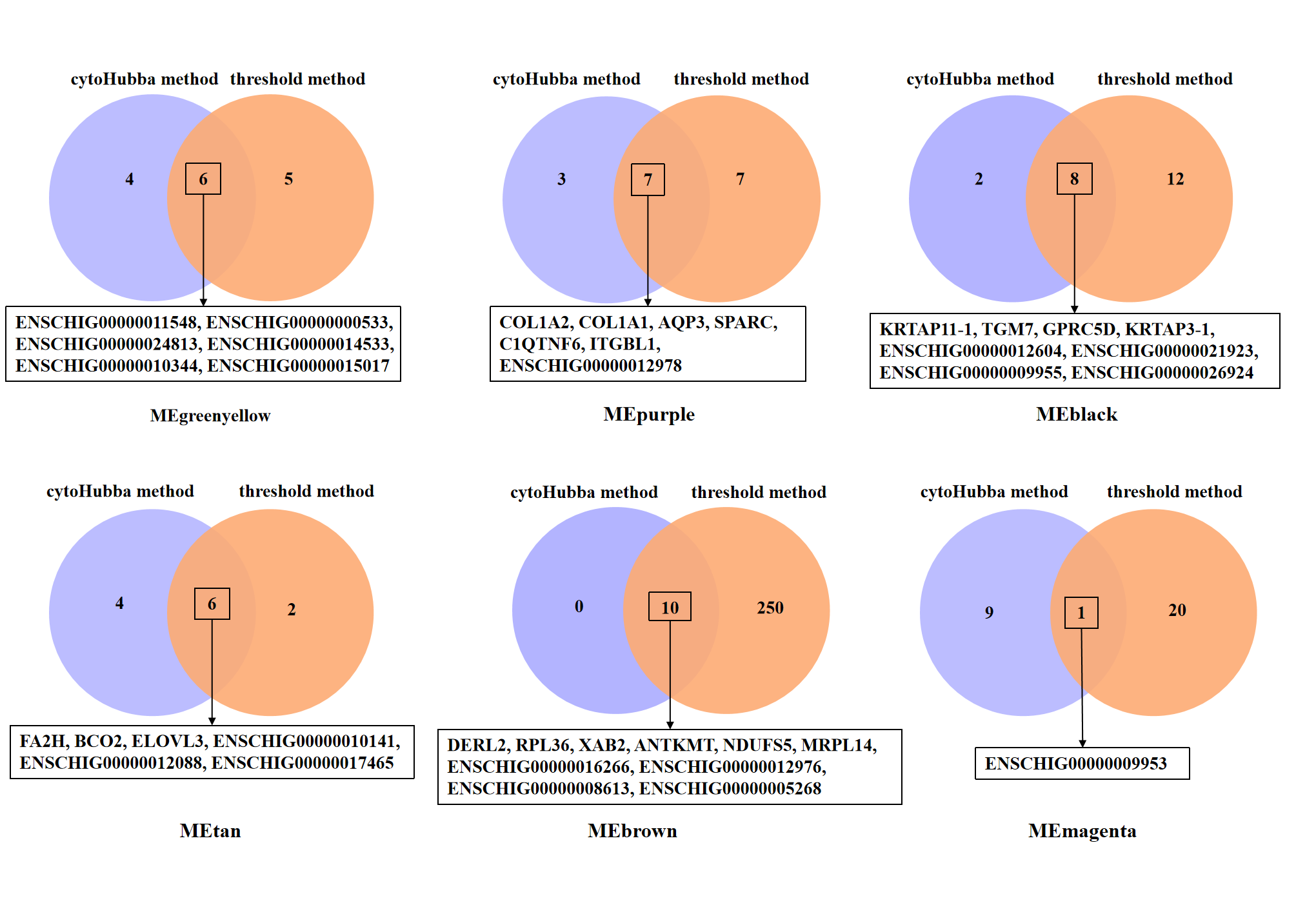

Supplement: Supplementary file 1 [file genes-12-00180-s001.zip › Supplemental Figure S4.png]
